# Detection and characterisation of multiple herpesviruses in free-living Western European hedgehogs (*Erinaceus europaeus*)

**DOI:** 10.1038/s41598-018-31900-w

**Published:** 2018-09-17

**Authors:** Helle B. Hydeskov, Akbar Dastjerdi, Kevin P. Hopkins, Marie-Pierre Ryser-Degiorgis, Frederik Widén, Andrew A. Cunningham, Becki Lawson

**Affiliations:** 1 Institute of Zoology, Zoological Society of London, Regents Park, London, NW1 4RY United Kingdom; 2 Veterinary Epidemiology, Economics and Public Health Group, Royal Veterinary College, Hawkshead Lane, North Mymms, Hatfield, AL9 7TA United Kingdom; 3 Animal and Plant Health Agency-Weybridge, Woodham Lane, Addlestone, Surrey, KT15 3NB United Kingdom; 4 Centre for Fish and Wildlife Health, Dept. Infectious Diseases and Pathobiology, Vetsuisse Faculty, University of Bern, Länggass-Str. 122, Postfach, 3001 Bern, Switzerland; 5 Department of Microbiology, National Veterinary Institute, 751 89 Uppsala, Sweden

## Abstract

Sporadic cases of herpesvirus-associated disease have been reported in the Western European hedgehog (*Erinaceus europaeus*), but there has been little surveillance for, nor any sequence characterisation of, herpesviruses in this species to date. A nested pan-herpesvirus polymerase chain reaction (PCR) targeting a region of the DNA polymerase gene was used to test 129 Western European hedgehogs from across Great Britain, 2011–2016; 59 (46%) of which were PCR-positive. In addition, samples from two previously published cases of fatal herpesvirus infection in *E. europaeus*, from Sweden and Switzerland, were positive using this PCR. No statistically significant relationship was detected between PCR result and sex, age class, year or season for the British hedgehogs tested. In most PCR-positive animals (19/22) from which liver and brain were tested separately, both were PCR-positive. Sanger sequencing of amplicons from 59 British hedgehogs revealed at least two novel viruses within the *Gammaherpesvirinae*. Thirteen of these hedgehogs had liver and brain tissues screened for microscopic abnormalities, of which one had non-suppurative meningoencephalitis, but neither intranuclear inclusion bodies nor herpesvirus virions (on electron microscopical examination) were identified. Sequencing of the whole DNA polymerase gene confirmed two genetically different *Human alphaherpesvirus 1* viruses in the Swedish and Swiss hedgehogs.

## Introduction

The Western European hedgehog (*Erinaceus europaeus*) is endemic to many European countries. Although it is listed as Least Concern by the IUCN Red List of Threatened Species^1^, it has been in significant decline for at least two decades in Great Britain^2,3^. Suggested causes for this decline include habitat loss and fragmentation, food limitation due to the usage of pesticides, and road traffic accidents^2,3^. It is unknown if infectious disease is contributing to this decline.

Whilst a range of infectious agents have been detected in hedgehogs^4^, investigations of viral aetiologies have typically been restricted to single case reports. Sporadic cases of herpesvirus-associated disease have been reported in the Western European hedgehog and the four-toed hedgehog (*Atelerix albiventris*)^5–8^. Herpesvirus-like particles were detected on transmission electron microscopy (TEM) examination of the liver of a free-living adult female Western European hedgehog that died soon after admission to a veterinary facility in the United Kingdom. Gross post-mortem findings included splenomegaly and a pale, mottled, friable and enlarged liver. Histopathological examination of the liver showed extensive areas of hyperaemia, parenchymal haemorrhage and hepatocellular necrosis with amphophilic intranucelar inclusion bodies within hepatocytes^5^. Herpesvirus-like particles were also observed on TEM examination of the liver of a three-month old female Western European hedgehog, held in captivity for hand rearing, which died with multifocal necrotising hepatitis in Sweden. In this case, virus isolation in primary bovine foetal skin cells revealed a cytopathic effect characteristic of an alphaherpesvirus infection. Gross findings in this hedgehog included a pale, yellow friable liver with petechial haemorrhages, splenomegaly, pulmonary oedema and an empty gastrointestinal tract^6^. *Human alphaherpesvirus 1* and *2* (genus *Simplexvirus*, subfamily *Alphaherpesvirinae*) antigen was visualised using immunohistochemistry in the brain of a young female Western European hedgehog in a Swiss rehabilitation centre. This animal, originally free-living, showed neurological clinical signs and died despite treatment. Gross findings included emaciation, enlarged prescapular lymph nodes and multifocal gastric ulcers. Histopathological examination showed mild aspiration pneumonia and severe meningoencephalitis with numerous eosinophilic intranuclear inclusion bodies in neurons and glial cells^7^. A similar alphaherpesvirus was detected in the liver of a captive adult female four-toed hedgehog (*Atelerix albiventris*) in the United States of America using virus isolation, immunolabelling and restriction fragment length polymorphism. The animal had a history of acute onset posterior ataxia with multifocal liver necrosis detected on post-mortem examination^8^. To our knowledge, no hedgehog herpesvirus has been genetically characterised to date.

The aims of this study were to investigate the occurrence of herpesvirus infections in free-living Western European hedgehogs in Great Britain, to characterise these herpesviruses and those previously detected, and to assess the clinical significance of the identified herpesvirus infections in this host.

## Methods

### Post-mortem examination and sample selection

A scanning surveillance scheme for the Western European hedgehog was conducted in Great Britain as part of the Garden Wildlife Health (GWH) project, coordinated by the Institute of Zoology^9^. Post-mortem examinations were performed on submitted hedgehog carcasses following a standardised protocol to ensure systematic inspection of all organ systems, as described by Franklinos *et al*.^10^. Each carcass was either examined upon arrival or was frozen at −20 °C pending examination. Age class was assigned based on dentition (deciduous versus permanent teeth). Where possible, a standard set of tissues, including liver and brain, was archived at −80 °C. Where the state of tissue preservation permitted, a standard set of tissues, plus any detected lesions, was fixed in neutral buffered 10% formalin. The date when, and location where, the animal was found dead, the age class and the sex were recorded for each hedgehog.

A convenience sample of liver and brain tissues from 129 free-living hedgehogs was included in the current study. Each hedgehog had been found dead or had been euthanased, 2011 to 2016. Cases with available liver and brain tissues were selected from the archive of 185 hedgehogs to ensure all years and geographical regions were represented. The majority (59%, n = 76) of these 129 hedgehogs were found dead by members of the public. The remainder were submitted by wildlife rehabilitation centres. Where information on duration of time in care was available, 82% (42/51) had died or had been euthanased within 48 hours of admission, therefore findings were presumed to reflect their health and infection status in the wild. Case selection was independent of the cause of death (i.e. including infectious and non-infectious disease), with the exception of a female juvenile hedgehog with non-suppurative meningoencephalitis. This was a juvenile female that had been held in captivity for treatment for several months in 2013, but which eventually was euthanased on welfare grounds. Post-mortem investigation identified localised jejunal cryptosporidiosis and non-suppurative meningoencephalitis, as previously described by Sangster *et al*.^11^. Samples from this hedgehog were included in the current study to investigate if herpesvirus infection was the cause of the non-suppurative meningoencephalitis.

In addition to the British hedgehogs, DNA extracted from the liver of a Swedish hedgehog^6^, and the brain of a Swiss hedgehog^7^, both previously diagnosed with alphaherpesvirus infection, were included in this study.

### DNA extraction

DNA extractions of the British hedgehog tissue samples were conducted using the DNeasy Blood & Tissue Kit (QIAGEN Ltd., Manchester, UK) following the manufacturer’s instructions. Equal weights of liver and brain tissues were pooled for DNA extraction from each of 128 hedgehogs. Liver and brain DNA were extracted separately from the hedgehog diagnosed with non-suppurative meningoencephalitis. Twenty-one of the hedgehogs which were polymerase chain reaction (PCR)-positive on pooled tissues subsequently also had DNA extracted from liver and brain separately.

DNA was extracted from frozen liver tissue from the Swedish hedgehog using 5% Chelex 100 Resin (Bio-Rad Laboratories Inc., Hercules, California, USA) and a 10% tissue suspension, according to the manufacturer’s instructions. DNA was extracted from formalin-fixed paraffin-embedded brain tissue from the Swiss hedgehog using the GeneRead DNA FFPE Kit (QIAGEN Ltd., Manchester, UK), according to the manufacturer’s instructions.

### Pan-herpesvirus PCR

A nested pan-herpesvirus consensus PCR protocol was used to amplify a region of the DNA polymerase gene of *Herpesviridae* (approximately 215–235 bp in length) using a mixture of ten degenerate and deoxyinosine-substituted primers^12^. The first round of PCR consisted of initial denaturation at 95 °C for 5 minutes followed by 40 cycles of denaturation at 95 °C for 20 seconds, annealing at 46 °C for 30 seconds and extension at 72 °C for 30 seconds and a final extension at 72 °C for 2 minutes. Each 20 µl reaction contained 10 µl QIAGEN Fast Cycling PCR Master Mix (2×) (Qiagen Ltd.), 0.5 µM of each first round forward and reverse primers and 2 µl template DNA. The second round of PCR consisted of initial denaturation at 95 °C for 5 minutes followed by 40 cycles of denaturation at 95 °C for 20 seconds, annealing at 46 °C for 20 seconds and extension at 72 °C for 20 seconds and a final extension at 72 °C for 2 minutes. The PCR mix was as above but contained second round forward and reverse primers and 1 µl of DNA. Amplicons from both the first and second round PCRs were visualised with gel electrophoresis using GelRed Nucleic Acid Gel Stain (Cambridge Bioscience Ltd., Cambridge, UK). Negative extraction controls (reagents only with no tissue to confirm absence of cross contamination during DNA extraction protocol), a negative PCR control (molecular grade water to confirm absence of cross contamination during the nested PCR protocol) and a positive control (DNA from *Bovine alphaherpesvirus 1*) were included in each PCR run. PCR-negative samples were repeated to exclude experimental error. The PCR protocol was run on all samples of extracted DNA (pooled and separate tissue extractions) from all the hedgehogs from Great Britain, as well as DNA extracted from the Swedish and Swiss hedgehogs. Two additional approaches were trialled in order to obtain further sequence data from the British samples: sequencing of first round pan-herpesvirus PCR products, and primers and protocol described by Ehlers *et al*.^13^ to amplify the glycoprotein B gene of as-yet-unknown gammaherpesviruses. At least one PCR product of the expected size was submitted to a commercial company (Genewiz UK Ltd., Bishop’s Stortford, UK) for clean-up and Sanger sequencing from each PCR-positive animal.

### Internal control PCR

Samples testing negative with the pan-herpesvirus PCR were subjected to an internal control PCR targeting a region of the host 16S rRNA gene (approximately 201–211 bp in length), using the protocol by Sarri *et al*.^14^. The PCR consisted of initial denaturation at 95 °C for 5 minutes followed by 35 cycles of denaturation at 95 °C for 40 seconds, annealing at 53 °C for 40 seconds, extension at 72 °C for 40 seconds and a final extension at 72 °C for 40 seconds. The PCR mix was as above but contained 1 µM of 16S rRNA forward and 16S rRNA reverse primers and 2.0 μl template DNA. PCR products were visualised as above. Three PCR products from the first PCR run were submitted to Genewiz UK Ltd. for clean-up and Sanger sequencing to confirm that they were DNA from a Western European hedgehog. One of these amplicons was then used as a positive control in each subsequent internal control PCR run.

### *Human alphaherpesvirus 1* DNA polymerase gene amplification

In order to genetically characterise the alphaherpesviruses reported from the Swedish and the Swiss hedgehogs, eight sets of overlapping primers targeting the entire DNA polymerase gene of *Human alphaherpesvirus 1* (approximately 4,300 bp) (Table 1) were designed using Primer3^15^. 2.0 μl DNA extracted from hedgehog tissue, and 0.5 µM of the *Human alphaherpesvirus 1* forward and reverse primers were used in the PCRs. The PCRs were carried out at 95 °C for 5 minutes for initial denaturation followed by 35 cycles of denaturation at 95 °C for 15 seconds, annealing at 55 °C for 15 seconds, extension at 72 °C for 1 minute and a final extension at 72 °C for 2 minutes. Where a primer pair failed to produce an amplicon in the PCRs, a 1:10 dilution of the PCR product was re-amplified following the initial PCR protocol. PCR products were visualised as above.

**Table 1:** PCR overlapping primer sets designed to amplify the entire DNA polymerase gene of hedgehog alphaherpesviruses.

Primer set no.	Forward primer	Reverse primer
1	5′ GTC CGG TAA TTT TGC CAT C 3′	5′ GTA CAC GTG AAA GAC GGT GAC 3′
2	5′ CAC GAC GGT CAC CTC AAG 3′	5′ AGT CGA AGT TGA TGA TGT TGT ACC 3′
3	5′ GAA TTC GAG ATG CTG TTG G 3′	5′ ATA GCT CAG GTC CTT CTT CTT G 3′
4	5′ GAT TAT AAC CGA CAA GAT CAA GC 3′	5′ AGT TAC ACA CGA CCT TGA TGG 3′
5	5′ AGA GAG CCT CCT CAG CAT C 3′	5′ CAA CAG GTG GGA GAA GTA ATA GTC 3′
6	5′ CTG CTG GTG TCC GAG CTG 3′	5′ ACA TTT ATT GTA AAA TGA GGG ACA TC 3′
7	5′ AAA AGG TTT ATT CCC GAA GTG 3′	5′ CAC ATG CTG TAC GTC ATC TTT C 3′
8	5′ TGA GCA TCC GGT CTA TGA G 3′	5′ ATG GGC TAC TAC CTA GGC ATC 3′

### Sequence analysis

Sequence editing was performed using Geneious version 7.1.9^16^. The primer-edited nucleotide sequences were initially compared with herpesvirus sequences in GenBank using BLASTN. DNASTAR Lasergene 13 (Lasergene®, Version 13, DNASTAR, Madison, WI, USA) was used to compare the similarity of the three British hedgehog herpesvirus sequences obtained.

### Pathological investigations

Gross post-mortem findings for liver and brain were reviewed for all pan-herpesvirus PCR-positive British hedgehogs. For the liver, special attention was given to lesions previously reported in association with herpesvirus infection in hedgehogs, including hepatomegaly, petechial haemorrhage and miliary or punctate foci in the liver^5,6,8^. Evidence of these abnormalities, or other evidence of substantial hepatic disease for which no alternative aetiology had been identified during the post-mortem examination and routine microbiological testing, was summarised. Although none of the previous reports of fatal herpesviral infections in hedgehogs^5–8^ identified any gross brain abnormalities, the brain gross post-mortem findings were reviewed for any substantial disease (e.g. congestion of brain vessels) for which no alternative aetiology had been identified during the post-mortem examination and routine microbiological testing. Thirteen PCR-positive hedgehogs for which there were well-preserved, formalin-fixed tissues available (i.e. excluding carcasses that had been frozen or which had undergone moderate or advanced autolysis) were selected for histopathological examination of the liver and brain. Formalin-fixed tissues were routinely processed for histological examination and stained with haematoxylin and eosin. Both tissue types were screened for the presence of intranuclear inclusion bodies, necrosis and inflammation; each of which has been previously reported not only in hedgehogs but also in other species affected by herpesviral disease^17^.

Transmission electron microscopy was conducted on frozen liver and brain tissue from the British hedgehog with meningoencephalitis using the method described by Everest *et al*.^18^.

### Data analysis

The proportion of PCR-positive hedgehogs was compared according to sex (male, female, unknown), age class (adults, juveniles, subadults, unknown), year found dead (2011–2016) and season found dead (winter, spring, summer, autumn) using Fisher’s exact tests calculated in RStudio (RStudio Desktop version 1.0.143)^19^. The significance threshold level was set at 0.05.

A map of the locations of the PCR-positive and PCR-negative hedgehogs was created using RStudio (RStudio Desktop version 1.0.143)^19^.

### Accession codes

All GenBank accession numbers are listed within the results.

## Results

### Pan-herpesvirus PCR

**Figure 1:**
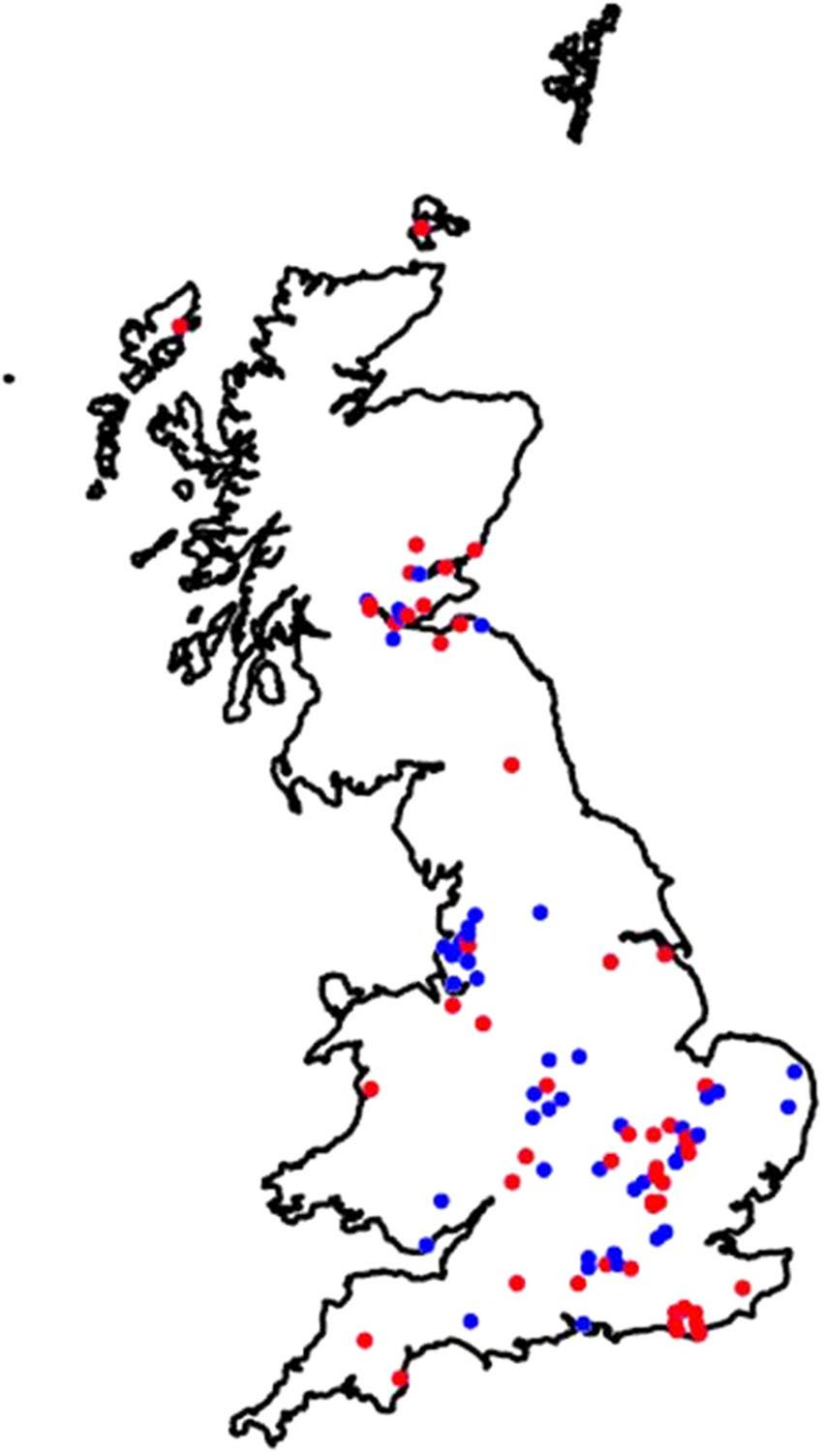
Geographical distribution of Western European hedgehogs (*Erinaceus europaeus*) subjected to the pan-herpesvirus PCR in Great Britain (n = 129). Red = PCR-positive hedgehog, blue = PCR-negative hedgehog. Locations with both PCR-positive and PCR-negative hedgehogs are shown as PCR-positive. The map was created using RStudio (RStudio Desktop version 1.0.143)^19^.

Of the 129 British hedgehogs examined, 59 (46%) were pan-herpesvirus PCR-positive. The numbers of PCR-positive and PCR-negative hedgehogs within the different groupings are shown in Table 2. There were no statistically significant relationships detected between PCR result and sex (p = 0.16), age class (p = 0.10), year (p = 0.76) or season (p = 0.29). The PCR-positive hedgehogs had a wide geographical distribution across Great Britain (Fig. 1). For the 22 hedgehogs for which liver and brain tissues were tested separately using the pan-herpesvirus PCR protocol, 19 (86%) were PCR-positive for both tissues, two (9%) were PCR-positive for the brain tissue only and one (5%) was PCR-positive for the liver tissue only. Both the Swedish and the Swiss hedgehog samples were pan-herpesvirus PCR-positive.

**Table 2:** Pan-herpesvirus PCR-positive percentage of Western European hedgehogs (*Erinaceus europaeus*) in Great Britain for different groups (*n* = 129).

Group	*n*	No. of PCR-positives	No. of PCR-negatives	PCR-positive percentage (%)	95% CI^a^ (%)
**Sex**					
Male	79	33	46	42	31–53
Female	48	26	22	54	39–68
Unknown	2	0	2	0	0–80
**Age class**					
Adult	70	39	31	56	43–67
Subadult	10	3	7	30	8–65
Juvenile	38	13	25	34	20–51
Unknown	11	4	7	36	12–68
**Year**					
2011	1	1	0	100	5–100
2012	24	11	13	46	26–67
2013	18	10	8	56	31–78
2014	34	15	19	44	28–62
2015	45	18	27	40	26–56
2016	7	4	3	57	20–88
**Season** ^**b**^					
Winter	16	9	7	56	31–79
Spring	31	16	15	52	33–69
Summer	39	13	26	33	20–50
Autumn	43	21	22	49	34–64
Total	129	59	70	46	37–55

^a^CI = confidence interval. ^b^Winter = December-February, spring = March-May, summer = June-August, autumn = September-November.

### Internal control PCR

All pan-herpesvirus PCR-negative samples were internal control PCR-positive for 16S rRNA. The three sequenced internal control PCR amplicons were identical (208 bp) and had 100% identity at 99% query coverage to a partial Western European hedgehog 16S rRNA gene sequence (GenBank accession number DQ630364.1).

### Herpesvirus identification

This study identified at least two gammaherpesviruses from the British hedgehogs, one alphaherpesvirus from the Swedish hedgehog and a second alphaherpesvirus from the Swiss hedgehog.

#### British hedgehog gammaherpesviruses

Sequencing of PCR amplicons from 57 of the 59 PCR-positive British hedgehogs revealed the same identical 166 bp primer-edited nucleotide sequence except for one single nucleotide polymorphism that did not code for a change in the amino acid sequence (GenBank accession number MG253640). These sequences (for the purpose of this study called Hedgehog gammaherpesvirus 1, HHGHV-1) had the highest similarity to *Myotis ricketti* herpesvirus 1 (GenBank accession number JN692429.1) with 84% identity at 43% query coverage.

Two different 166 bp nucleotide sequences (for the purpose of this study called Hedgehog gammaherpesvirus 2a and 2b, HHGHV-2a and HHGHV-2b) were obtained from the two remaining PCR-positive British hedgehogs for which the PCR amplicon was sequenced (GenBank accession numbers MG599085 and MG253639, respectively). HHGHV-2a and HHGHV-2b differed from each other by one non-synonymous substitution (threonine to isoleucine), confirmed by repeated PCR and sequencing, and were 99.4% similar at the nucleotide level for the genomic region sequenced. The sequences had highest similarity to 13 bat herpesviruses (e.g. GenBank accession number KR261894.1) with approximately 81% identity at 40% query coverage.

HHGHV-2a and HHGHV-2b shared 55.9–56.5% nucleotide similarity with the HHGHV-1 for the regions sequenced. The PCRs using either the first round primers of the pan-herpesvirus PCR or the primers described by Ehlers *et al*.^13^ for the glycoprotein B gene of gammaherpesviruses did not produce an amplicon of the correct size or a gammaherpesvirus sequence.

#### Alphaherpesvirus

The primer-edited nucleotide sequences obtained for the pan-herpesvirus PCR amplicons from the Swedish and Swiss hedgehogs were 178 bp long (GenBank accession number MG253638), identical to each other and to those of *Human alphaherpesvirus 1* (e.g. GenBank accession number NC_001806.2).

The complete DNA polymerase gene sequence was obtained for the Swedish, but not for the Swiss, sample. The DNA polymerase gene sequence for the Swedish hedgehog herpesvirus was 3708 nucleotides coding for 1235 amino acids (GenBank accession number MG253637). The PCR amplification and sequencing of the Swiss hedgehog herpesvirus concluded in three disjointed contigs of 429, 299 and 286 (GenBank accession numbers MG253635, MG253636 and MG253634, respectively) nucleotides having up to three mismatches compared to sequences of corresponding regions on the Swedish hedgehog herpesvirus DNA polymerase gene. The Swedish hedgehog herpesvirus DNA polymerase gene sequence differed from that of *Human alphaherpesvirus 1* by two non-synonymous substitutions (threonine to proline). A nucleotide mismatch in the 286 nucleotide segment of the Swiss hedgehog herpesvirus also resulted in a non-synonymous substitution (threonine to isoleucine), compared to *Human alphaherpesvirus 1*. Collectively, these findings confirm that the Swedish and Swiss hedgehogs were both infected with *Human alphaherpesvirus 1*.

### Pathological investigations

No gross abnormalities consistent with herpesvirus-associated hepatic or brain disease, and for which no alternative aetiology had been identified, were found in the review of the 59 pan-herpesvirus PCR-positive hedgehogs from Great Britain. On histopathological examination, abnormalities were detected in two hedgehogs: The animal with non-suppurative meningoencephalitis^11^; and another animal which had a few multinucleated hepatocytes in the liver. No evidence of inclusion bodies were observed in any section examined. TEM performed on the liver and brain tissues of the hedgehog with meningoencephalitis failed to detect any virions or any other causative agent.

## Discussion

In this study, we provide evidence that the Western European hedgehog, like other vertebrate species^20^, is susceptible to infection with multiple herpesviruses, including at least two novel gammaherpesviruses. There was no apparent predisposition to infection by sex, age class, year or season. In Great Britain, the PCR-positive hedgehogs had a wide spatial distribution with no apparent evidence of geographical clustering, except that expected from a convenience sample (e.g. increased submissions from areas local to a wildlife rehabilitation centre). These findings indicate that HHGHV-1 is endemic in the free-living hedgehog population in Great Britain, with infection being spatio-temporally widespread at moderately high occurrence. In contrast, HHGHV-2a and HHGHV-2b were each found in only one hedgehog and whether these viruses are also endemic in the British hedgehog population requires examination and sequencing of HHGHVs from a larger sample of hedgehogs across Great Britain.

The sequences obtained for the British hedgehog gammaherpesviruses were relatively short and therefore limited phylogenetic characterisation beyond subfamily level. Sequencing of the entire DNA polymerase gene would be beneficial for further characterisation. Unfortunately, as the genomes of these gammaherpesviruses are still unknown there are no sequence data available for further primer design. The first round of pan-herpesvirus PCR failed to produce a detectable product, possibly indicative of low virus concentration in the examined tissues. PCR and sequencing of additional genes, as conducted in other studies^21,22^, is likely to be unsuccessful as evidenced by the outcome of our attempts to use the primers described by Ehlers *et al*.^13^. Random amplification of the extracted DNA followed by whole genome sequencing would be an alternative option for further characterisation of the British hedgehog gammaherpesviruses.

The clinical significance of the British hedgehog gammaherpesviruses detected in this study remains uncertain. Whilst meningoencephalitis was observed in one hedgehog infected with HHGHV-1, no inclusion bodies were observed on microscopic examination nor were any virions detected using TEM; therefore, it remains unclear if HHGHV-1 was the causative agent of this disease. Furthermore, none of the other 58 British pan-herpesvirus PCR-positive hedgehogs had any gross post-mortem findings related to herpesvirus infection and none of the 12 PCR-positive hedgehogs examined microscopically had any lesions indicative of herpesviral disease^5–8,17^. We suggest, therefore, that HHGHV-1 infection (and potentially also HHGHV-2a and HHGHV-2b infections) were subclinical in the hedgehogs we examined. Whether these viruses can cause substantial disease on initial infection or when reactivated, such as when the immune system is compromised, as can occur with some alpha-, beta- and gammaherpesvirus infections in humans^23^, remains unknown. This result from our study is not surprising. Although some gammaherpesvirus infections have been associated with disease in primates (e.g. *Human gammaherpesvirus 4* causing mononucleosis in people)^24^ and in ungulates (e.g. *Ovine gammaherpesvirus 2* and *Caprine gammaherpesvirus 2* causing malignant catarrhal fever in ungulates)^25^, other gammaherpesvirus infections have not been associated with disease in other mammalian species^21,26^.

The availability of well-preserved, formalin-fixed tissues limited the number of hedgehogs examined histologically in this study. Further work is required to look for evidence of disease associated with HHGHVs. In addition to histological examination of a larger sample size, the use of techniques to visualise the presence of viral DNA or virions, and explore their co-occurrence with any lesions found, would further enable assessment of causality. Potential options include the development of an *in situ* hybridisation protocol^27^ based on available sequence data or the use of TEM^5,6^. The development of HHGHV-1-, HHGHV-2b- and HHGHV-2b-specific qPCRs would enable viral loads to be compared between tissues which might help infer potential sites of viral replication and/or persistence.

The identification of *Human alphaherpesvirus 1*, the most common causative agent of human cold sores^28^, in both the Swedish and Swiss hedgehogs is of interest. In addition to being able to naturally infect and cause encephalitis in the domestic rabbit (*Oryctolagus cuniculus*)^29^, it appears that *Human alphaherpesvirus 1* is also able to infect and cause disease (hepatitis or meningoencephalitis) in the Western European hedgehog. Although this virus was not proved to have caused disease in the Swedish and Swiss hedgehogs, the presence of viral particles was closely associated with the observed lesions and such a causal relationship seems likely. It should be noted that both of these hedgehogs were held in captivity at wildlife treatment and rehabilitation facilities, therefore there was opportunity for close contact with people which is unlikely to occur with free-living hedgehogs. Our results highlight the anthroponotic potential of this virus and the need for people working with this species to practice good hygiene and biosecurity.
